# Ultrasonic osteotomy and regeneration with A-PRF+ for periapical surgery of a mandibular molar with root cyst perforating the lingual osseous cortex: A case report

**DOI:** 10.4317/jced.60830

**Published:** 2023-09-01

**Authors:** Vicent Sanz-Zornoza, Juan-Carlos Bernabeu-Mira, David Soto-Peñaloza, David Peñarrocha-Oltra, Miguel Peñarrocha-Diago

**Affiliations:** 1 Grade of Dentistry, Faculty of Medicine and Dentistry, Universitat de València, Valencia, Spain 2 Master in Oral Surgery and Implantology, Faculty of Medicine and Dentistry, University of Valencia, Valencia, Spain; 2 Master in Oral Surgery and Implantology, Faculty of Medicine and Dentistry, University of Valencia, Valencia, Spain; 3 Professor of Oral Surgery, Director of Master in Oral Surgery and Implantology, Faculty of Medicine and Dentistry, University of Valencia, Spain. Researcher at IDIBELL, Barcelona. Spain; 4 Professor of Oral Surgery, Faculty of Medicine and Dentistry, University of Valencia, Valencia, Spain. Researcher at IDIBELL, Barcelona. Spain

## Abstract

**Background:**

Periapical surgery is a complex dental procedure that remains a considerable challenge in clinical practice. The use of APRF+ membranes and piezoelectric osteotomy help to improve outcomes and increase the efficiency and speed of recovery.

**Material and Methods:**

This case report describes a 20 years-old man with a periapical lesion which perforated the lingual cortex in a lower mandibular molar. A periapical surgery was performed with endoscope magnification and ultrasonic osteotomy. Apicectomy and retrograde cavities were performed using a piezoelectric scalpel and sealed using a bioceramic sealer. The osteolytic defect was filled with A-PRF+ membranes and the bone cortex was repositioned trough a micro-screw.

**Results:**

The histological analysis concluded an inflammatory odontogenic cyst. The postoperative period was uneventful with pain and mild oedema until the fourth day. Short-term follow-up showed the beginnings of bone regeneration and correct healing of the surgery without periodontal defects. Two-year follow-up showed favorable results and regeneration of the bone defect.

**Conclusions:**

Periapical surgery with magnification, ultrasonic osteotomy repositioning and application of A-PRF+ membranes as an adjuvant proved to be an effective approach for the regeneration of the osteolytic process, allowing the preservation of the tooth. Promising short and long-term results were shown for this case report.

** Key words:**Periapical surgery, osteotomy, ultrasound, A-PRF+.

## Introduction

The incidence of inflammatory root cysts is around 55% of the total number of odontogenic cysts in the jaws (1).

Current surgical techniques, supported by the use of ultrasound devices, magnification (2,3) and the use of new bioactive cements such as TotalFill™, have improved the prognosis of periapical surgery.

When there is a higher risk of incomplete healing, the use of guided tissue regeneration has been described with the aim of maintaining space in the bone cavity, stabilization of the clot and migration of bone-forming cells (4). In addition, the use of membrane-based platelet concentrates, such as advanced platelet-rich fibrin (A-PRF+) (5), has been proposed as an alternative to stabilize the clot in irregularly shaped lesions and its use in periapical surgery has been associated with an improved postoperative quality of life (6,7).

The purpose of this article is to describe the treatment of a mandibular first molar with a periapical lesion perforating the lingual cortex by periapical surgery performed with ultrasonic tip osteotomy and filling of the defect with A-PRF+ membranes as an adjuvant to healing.

## Case Report

**Figure 1:**
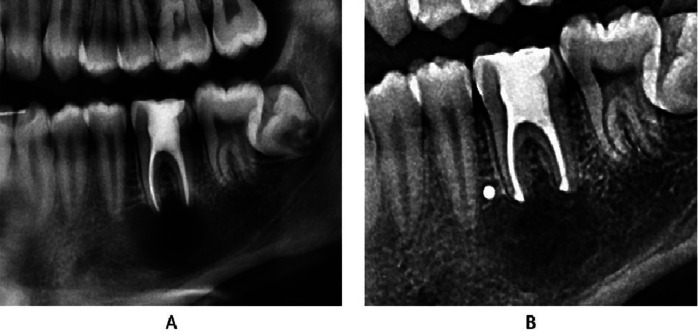
Orthopantomography images. A: Preoperative orthopantomography. Apical radiolucent zone of first mandibular molar was detected. B: Control orthopantomography 6 months after surgery. Bone regeneration can be detected.

A 20-year-old man with no medical history of interest or known allergies, underwent a routine orthopantomography (PLANMECA ProMax 3D Classic, Planmeca Oy, Helsinki, Finland). A large radiolucent lesion was found by chance in the apical area of the left first mandibular molar (Fig. 1A). The patient reported no symptoms. The tooth did not show mobility or pain on percussion, and it was periodontally healthy. The affected tooth was not vital, but positive vitality of the adjacent teeth was verified by means of cold stimuli.

**Figure 2:**
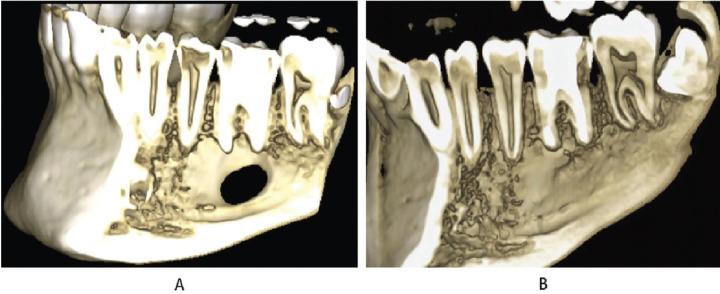
Three-dimensional reconstruction of the CBCT. A: Before the surgery. Lingual cortical loss can be detected. B: 2 years follow up showed the lingual cortical completely regenerated.

A cone beam computed tomography (CBCT) scan (PLANMECA ProMax 3D Classic, Planmeca Oy, Helsinki, Finland) was performed to plan the surgery. The CBCT showed a perforation of the lingual cortex (Fig. 2A). The patient signed the informed consent for the treatment and for the use of the data with scientific purposes.

Obtaining of A-PRF+: Prior to the start of surgery, the median cubital vein was punctured to collect three 10 mL glass tubes without additives (Plain Vacuum Tube, A-PRF+, Process for PRF, Nice, France). The tubes were centrifuged at 1300 rpm (208 g) for 8 minutes (A-PRF DUO Centrifugate, Process for PRF, Nice, France). The fibrin clots obtained were extracted with special forceps and scissors, avoiding the red component of the blood under the leukocyte layer, and subsequently processed to obtain a standardized membrane thickness in a sterile container (PRF-BOX, Process dor PRF, Nice, France).

Surgical procedure: Truncal anesthesia (articaine 4% (1:100,000) (Inibsa; Lliça de Vall, Barcelona, Spain)) of the lower dental and buccal nerve and subperiosteal infiltrations were performed in the surgical area.

**Figure 3:**
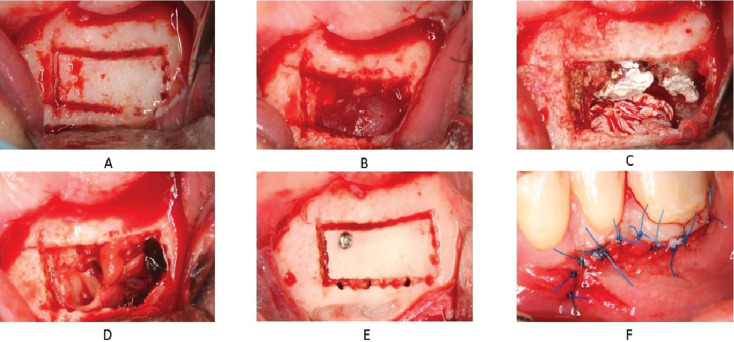
Clinical images. A: The grooves made with an ultrasonic tip and drilled with a fissure drill can be detected. B: The bone plate has been removed exposing the lesion under the cortex. C: The operated cavity. Here the roots can be showed with the TotalFill filling material. D: Filling of the cavity with A-PRF membranes. E: Detail of bone plate screwed with osteosynthesis screw. F: Detail of the single stitch suture.

After elevation of a full-thickness submarginal flap, an osteotomy procedure (7) was chosen. Using ultrasonic tips (*Pi*ezomed; W&H Dentalwerk, Bürmoos, Austria) and a fissure reamer (Fig. 3A), the bone plate was removed with a chisel and hammer and the periapical lesion was exposed (Fig. 3B).

The lesion was carefully excised. All excised material was collected and kept in 10% formalin for a histological examination. Subsequently, curettage of the cavity was completed.

After exposing the apices, the apical portion of the roots was resected and a 3-mm deep retrograde cavity was instrumented with ultrasonic tips (*Pi*ezomed, W&H Dentalwerk, Bürmoos, Austria). Hemostasis was achieved using sterile polytetrafluoroethylene strips (8). The retrograde cavity was dried with sterile paper tips and obturated with a bioceramic root repair material (TotalFill; Innovative BioCeramix Inc., North Fraser Way Burnaby, BC, Canada) (Fig. 3C).

After removal of the remaining retrograde filling material from the root surface and bone crypt, autologous advanced platelet-rich fibrin (A-PRF+) membranes obtained from the patient’s blood samples were placed as a regeneration adjuvant (Fig. 3D).

The previously extracted bone plate was positioned and an osteosynthesis screw (Meisinger Screw System TX, Hager&Meisinger, Neuss; Germany) was inserted for better fixation and to avoid any displacement (Figure 3E). Finally, the flap was approached to the attached gingival edges for suturing. This was done using 11 single stitches with non-absorbable monofilament suture (Seralene, Serag-Weissener GmbH & Co. KG®, Naila, Germany) (Fig. 3F).

An orthopantomography and a periapical radiograph were performed after completion of the operation to check the apical seal and to evaluate the evolution of the area in order to assess its recovery.

Postoperative: Postoperative care was indicated, and the patient was prescribed Amoxicillin 500mg (Amoxicillin Cinfa, Laboratorios Cinfa, S.A., Huerta Pamplona, Navarra, Spain) 1 Tablet every 8 hours and Ibuprofen 600mg every 8 hours, all for 7 days.

## Results

Anatomo-pathological diagnosis: the fragments analyzed were fibrocellular connective tissue with variable density and intense inflammatory infiltrate of a chronic nature, with accumulations of foamy macrophages, polystratified, non-keratinised, hyperplastic material. The conclusion was that it was an inflammatory odontogenic cyst resulting from possible apical accessory canals as the conventional root canal treatment had been performed correctly.

The patient was seen one week later for removal of stitches and an optimal evolution of the soft tissue healing was observed. The patient presented slight oedema in the area of the operation for 4 days without hard pain or discomfort. There was also no hematoma or bleeding as result of the operation. There was no gingival recession related to the surgery.

A radiographic control was performed at 6 months where partial regeneration of the bone tissue was observed (Fig. 1B). At two years, the surgery was checked and a control CBCT showed satisfactory results with almost complete bone regeneration (Fig. 2B) (9).

## Discussion

In this clinical case, periapical surgery with osteotomy repositioning and A-PRF+ membrane regeneration was performed on a mandibular first molar. Osteotomy versus ostectomy may result in a better postoperative period with less pain and swelling (7). *Pi*ezoelectric tips allow the creation of precise bone windows and the subsequent repositioning of bone cortex. The cortex acts as a barrier providing support and stability to the cavity, hindering the invagination of connective and epithelial tissue cells. (10).

A preclinical study in sheep demonstrated greater effectiveness of cortical repositioning versus cavity sealing with other materials in maxillary sinus elevations, observing a greater amount of neoformed bone (11). In addition, cortical bone repositioning has been found to aid in the resolution of traumatic osteolytic processes (12) very similar to the present case.

Von Arx and AlSaedd M. classified periapical lesions into three types to analyze the need for bone regeneration: delimited apical lesions without cortical involvement, “tunnel” lesions with perforation of the osseous cortices and defects with apicomarginal involvement (10). In the case of tunnel lesions, they concluded that the use of guided regeneration can be useful to avoid excessive migration of soft tissue and favor the formation of bone tissue (10). In the present case, the use of A-PRF+ was proposed to improve healing by stabilizing the coagulum (13,14) and its use in periapical surgery has been associated with improved postoperative quality of life (6).

Regarding magnification, the use of an endoscope allows correct magnification and illumination to perform endodontic surgery and is associated with high success rates (15).

Histological examination confirmed the diagnosis of an inflammatory odontogenic cyst. A clinical case report has low scientific quality, so larger studies are needed to determine if A-PRF+ and cortical repositioning aid bone regeneration in periapical surgery.

## Conclusions

Periapical surgery with magnification, ultrasonic osteotomy repositioning and application of A-PRF+ membranes as an adjuvant proved to be an effective approach for the regeneration of the osteolytic process, allowing the preservation of the tooth. Promising short and long-term results were shown for this case report. More clinical studies, specially randomized clinical trial, are necessary.
